# Novel nanographene oxide-calcium phosphate cement inhibits *Enterococcus faecalis* biofilm and supports dental pulp stem cells

**DOI:** 10.1186/s13018-021-02736-4

**Published:** 2021-10-09

**Authors:** Shizhou Wu, Michael D. Weir, Lei Lei, Jun Liu, Hockin H. K. Xu

**Affiliations:** 1 Department of Orthopedic Surgery, West China Hospital, Sichuan University, Chengdu, 610041 Sichuan China; 2 Biomaterials & Tissue Engineering Division, Department of Advanced Oral Sciences and Therapeutics, University of Maryland Dental School, Baltimore, MD 21201 USA; 3 State Key Laboratory of Oral Diseases & National Clinical Research Center for Oral Diseases, West China Hospital of Stomatology, Sichuan University, Chengdu, 610041 Sichuan China; 4 Marlene and Stewart Greenebaum Cancer Center, University of Maryland School of Medicine, Baltimore, MD 21201 USA; 5 Center for Stem Cell Biology and Regenerative Medicine, University of Maryland School of Medicine, Baltimore, MD 21201 USA

**Keywords:** Calcium phosphate, Graphene oxide, Injectable, Antibacterial, *Enterococcus faecalis*, Stem cells

## Abstract

**Background:**

*Enterococcus faecalis* (*E. faecalis*) is the most recovered species from the root canals after failed root canal treatment. Calcium phosphate bone cement (CPC) scaffold is promising for applications in endodontic treatment as a kind of root canal sealer. Graphene oxide (GO) has been extensively considered as a kind of promising nano-materials for antibacterial applications. In the present study, an injectable CPC-chitosan paste containing GO was developed for promising endodontic therapy. The antibacterial properties of this paste against *E. faecalis* biofilms as well as the support for human dental pulp stem cells (hDPSCs) were investigated.

**Methods:**

CPC-chitosan composite with or without GO injectable scaffold was fabricated. The hDPSC growth and viability on scaffolds were investigated by live/dead assay. Antibacterial effects against *E. faecalis* biofilms were determined in clinical detin block samples.

**Results:**

The antibacterial CPC-chitosan-GO disks had excellent hDPSC support with the percentages of live cells at around 90%. CPC-chitosan-GO also had greater antibacterial activity on *E. faecalis* than that of CPC-chitosan control using detin block models (*p* < 0.05).

**Conclusions:**

The injectable CPC-chitosan-GO paste had strong effects on inhibition *E. faecalis* and hDPSC support, which could fill the void of adjusting paste to the defect and shaping in situ for promising endodontic therapy.

## Introduction

Root canal therapy (RCT) is an essential step to remove infected tissue and pathogens such as *Enterococcus faecalis* (*E. faecalis*), one of the most recovered species from the root canals after failed root RCT [1]. For goals of RCT, the suitable filling materials involving sealing ability, biocompatibility, and antibacterial properties are supposed to be applied to occupy the root canal systems with anatomical complexity [2]. However, the resistance of *E. faecalis* to the medicament and filling materials has been consequently demonstrated [3]. Calcium phosphate cement (CPC) is a kind of bone mineral-mimicking apatite containing tetracalcium phosphate (TTCP) and dicalcium phosphate-anhydrous (DCPA). It is promising to be applicated in endodontic treatment as root canal sealer [4]. Our previous studies indicated that the mechanical properties of CPC could be enhanced by incorporation of chitosan, which may make CPC-chitosan paste more suitable for in situ repairs with injectability and bioactivity [5].

Recently, nanomaterials such as graphene oxide (GO) are considered as effective alternative antimicrobial agents for antibiotics and chemical agents [6]. The sharp edges of GO nanosheets could be as a “nano knife” resulting in physical damages for bacterial membrane integrity which causes the ROS synthesis for the antibacterial activity [7]. A wide range of antibacterial properties such as *E. coli* and *Pseudomonas aeruginosa* were verified [8]. Due to the unique properties including large surface planar structure, chemical and mechanical stability, and good biocompatibility, GO has been extensively considered as a kind of promising biomaterials for antibacterial applications^8^. Our previous in vitro study identified CPC-chitosan-GO antibacterial potential for *Staphylococcus aureus* [9].

Stem cell-based therapy is a promising strategy to repair injured lesions for the tissue regeneration [10]. Human dental pulp stem cells (hDPSCs) are kind of neural crest-related stem cells, which can be isolated from human dental pulp tissues [11]. Compared to bone marrow mesenchymal stem cells (BMSCs), which often regarded as the gold standard for mesenchymal stem cells (MSC) research, the DPSCs, with the large harvest of cells, demonstrate a higher proliferation and differentiation ability, which could interact with biomaterials and provide even greater pulp regeneration capacity [12].

Ideally disinfection methods should be sought that do not affect the dentin structure and support stem cell differentiation. Graphene oxide (GO), with good biosafety, was observed by co-culturing with BMSCs and implanting materials into mice muscle tissue [13]. However, the possibility of this novel CPC-chitosan-GO paste application on *E. faecalis* attributed failed root canal endodontic therapy and supportability for hDPSCs prepared for regenerative endodontics were elusive. In the present study, an antibacterial and injectable CPC-chitosan paste containing GO was developed for the potential application in the endodontic therapy. A clinical detin model consisting the clinical isolated strain was adopted to confirm the antibacterial properties of this injectable CPC-chitosan-GO paste. The aims of this study were to investigate the antibacterial properties against *E. faecalis* biofilms as well as the support for hDPSCs. The following hypotheses were tested: (1) the injectable CPC-chitosan paste with GO could provide a reliable antimicrobial therapy against *E. faecalis* biofilms; and (2) the injectable CPC-chitosan-GO scaffold would have no toxic effects and would support hDPSC viability.

## Materials and methods

### Fabrication of CPC-chitosan composite with or without Graphene oxide (GO)

The CPC powder was made by mixing and milling tetra-calcium phosphate (TTCP) (Ca_4_ (PO_4_)_2_O) and anhydrous dicalcium phosphate (DCPA) (CaHPO_4_) as previously described. Then 7.5% chitosan (Halosource Inc., Redmond WA) solution was stirred homogeneously with CPC power at a ratio of 2:1 in mass to obtain CPC-chitosan paste. For CPC-chitosan-GO paste, the GO nano powder (XFNANO Materials Tech, Nanjing, China) was mixed with the CPC-chitosan paste to obtain a final GO concentration of 50 μg/mL. The composite disks were prepared as previously described in specific molds [9].

### hDPSCs proliferation and cell viability on CPC-chitosan composite disks for biocompatibility test

hDPSCs were isolated and characterized as described previously from healthy human adult third molars [14]. The procedure was approved by the Ethical Committee of the University (NO. KS2020446). DPSCs were identified by positively expressed surface markers of MSCs (CD29, CD44, CD166, and CD73) and negative typical hematopoietic markers (CD34, CD45, and CD14) [15]. Cells at 4–5 passages were used for the present study.

According to our previous study [9], the capacity of CPC-chitosan scaffold with or without GO for hDPSCs support was determined by live/dead assay for 1 day, 3 days, and 5 days culture. The percentage of live hDPSCs were examined and calculated for stem cell support. Three groups were tested: (1) CPC-chitosan group (CPC + 7.5% chitosan liquid + hDPSCs); (2) CPC-chitosan-GO group (CPC + 7.5% chitosan liquid + GO powder + hDPSCs); and (3) blank control group.

### *E. faecalis* culture conditions and antimicrobial property test for *E. faecalis* biofilm on CPC-chitosan disks with or without GO

The *E. faecalis* standard strain V583 was cultured in brain–heart infusion medium (BHI; Becton Dickinson, Franklin Lakes, NJ). Sterile CPC-chitosan disks with or without GO were placed in 24-well polystyrene culture plates with *E. faecalis* to established 24 h biofilms. Three groups were tested for antibacterial properties: (1) CPC-chitosan group (CPC + 7.5% chitosan liquid + 24-h *E. faecalis biofilm*); (2) CPC-chitosan-GO group (CPC + 7.5% chitosan liquid + GO powder + 24-h *E. faecalis biofilm*); and (3) the blank control group.

The biofilm was labeled with SYTO9 and propidium iodide (Invitrogen, Carlsbad, CA, USA) and vitality was assessed by with epifluorescence microscopy (Nikon Eclipse TE-2000S, Melville, NY). For inhibition zone assay**,** the CPC-chitosan and CPC-chitosan-GO disks were placed in the center of the *E. faecalis* spread BHI agar plates respectively, and the inhibition zones around the disk samples were measured after one-day incubation at 37 ℃ in 5% CO_2_. In addition, colony-forming units (CFU) assays were adopted for quantitative analysis on the CPC-chitosan disks with or with GO for anti-biofilm testing. Twenty four-hour biofilms on the CPC-chitosan disks with or with GO were placed were immersed in 1 mL of sterilized phosphate-buffered saline for 10-min ultrasonic bath. Then the suspensions were diluted and dropped into BHI agar plates for 24-h incubation at 37 ℃ and the number of colonies grown on each plate was calculated [16].

### Antimicrobial property test for *E. faecalis* biofilm on CPC-chitosan paste with or without GO on clinical detin block samples

**Fig. 1:**
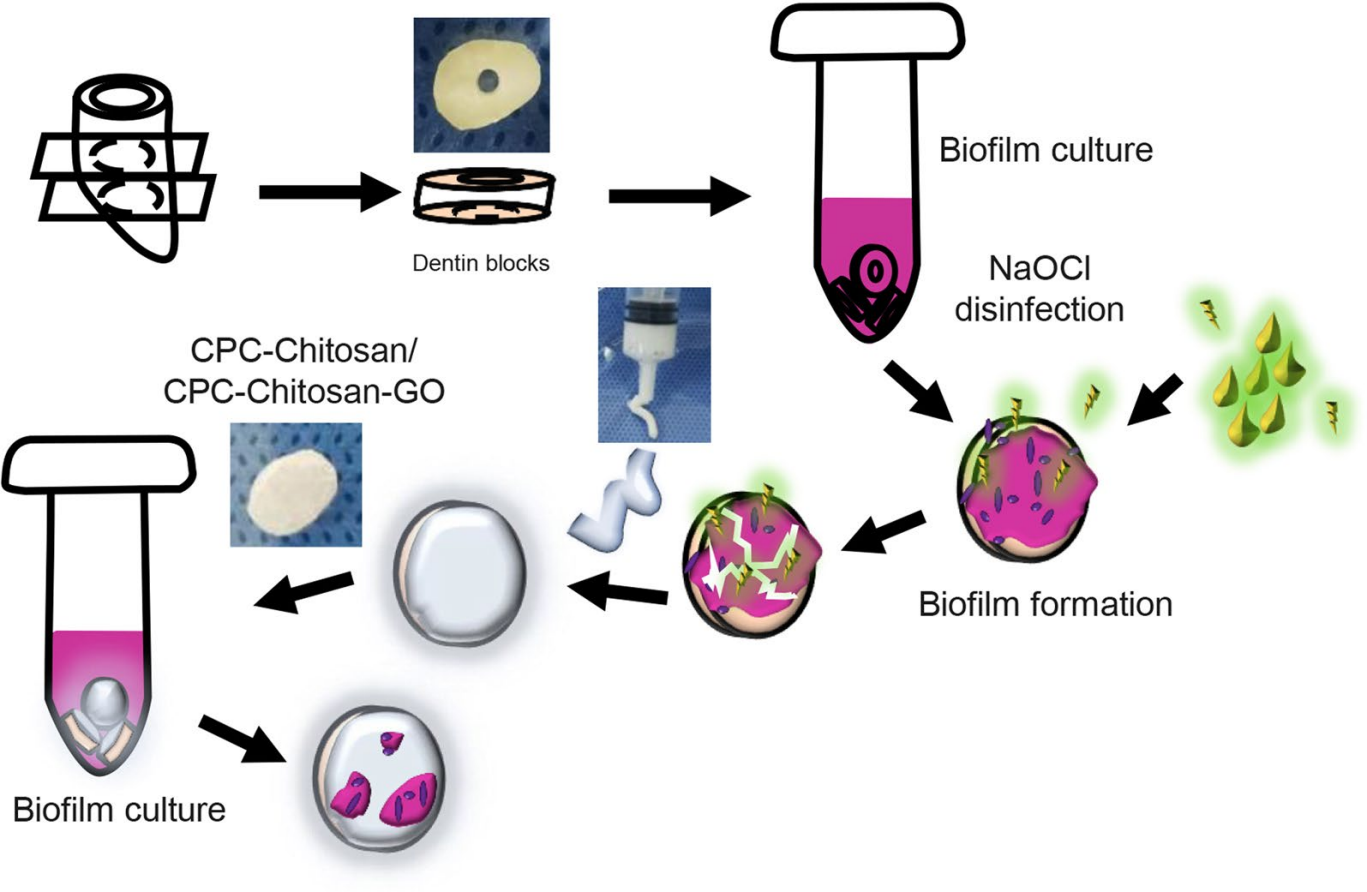
Schematic drawings of dentinal sample preparation. The dentin blocks were prepared by dental handpiece and immersed into mid-phased *E. faecalis* suspension for 24 h to acquire infective dentin blocks. Then ultrasonic bath in the 1% sodium hypochlorite solution was applied to resolve constructed biofilms on infective dentin blocks. Thereafter CPC-chitosan or CPC-chitosan-GO paste was intruded to cover the surface of disinfected dentin blocks as a kind of sealer and the dentin samples were immersed again into mid-phased *E. faecalis* suspension for 24 h to investigate their antibacterial property

Clinical teeth samples were collected as previous described [17]. The isolated clinical *E. faecalis* strain was inoculated onto Pfizer selective agar plates (Huankai, Guangzhou, China) and selected by colony morphology, Gram staining, oxygen tolerance, bile resistant and 16S rRNA [18]. The infective dentin specimens were processed as shown in Fig. 1. Three groups were tested for antibacterial properties: (1) CPC-chitosan group (CPC + 7.5% chitosan liquid + 24-h clinical isolated *E. faecalis* biofilm); (2) CPC-chitosan- GO group (CPC + 7.5% chitosan liquid + GO power with 0.05% w/v in the scaffold + 24-h clinical isolated *E. faecalis* biofilm); (3) dentin block group.

Scanning electron microscopy (SEM, Quanta 200, FEI, Hillsboro, OR, USA) and confocal laser scanning microscope (CLSM, FV1000; Olympus Corporation, Tokyo, Japan) were applied to evaluate the clinical isolated *E. faecalis* biofilm on CPC-chitosan paste with or without GO on clinical detin block samples. For SEM observation, the biofilms on disks were fixed with 2.5% (v/v) glutaraldehyde overnight. Then, the samples were experienced a sequential dehydration in ethanol solutions and then were prepared for SEM imaging. In addition, colony-forming units (CFU) assays were adopted for quantitative anti-biofilm testing as described above.

### Statistical analysis

Statistical analyses were performed using Statistical Package for the Social Sciences (SPSS 19.0, Chicago, IL, USA). One-way ANOVA analysis followed by post hoc LSD (least significant difference) tests or Student's t test was proceeded to detect the significant differences of the variables. All statistical analysis was considered significant when *p* ≤ 0.05.

## Results

### Stem cell viability

**Fig. 2:**
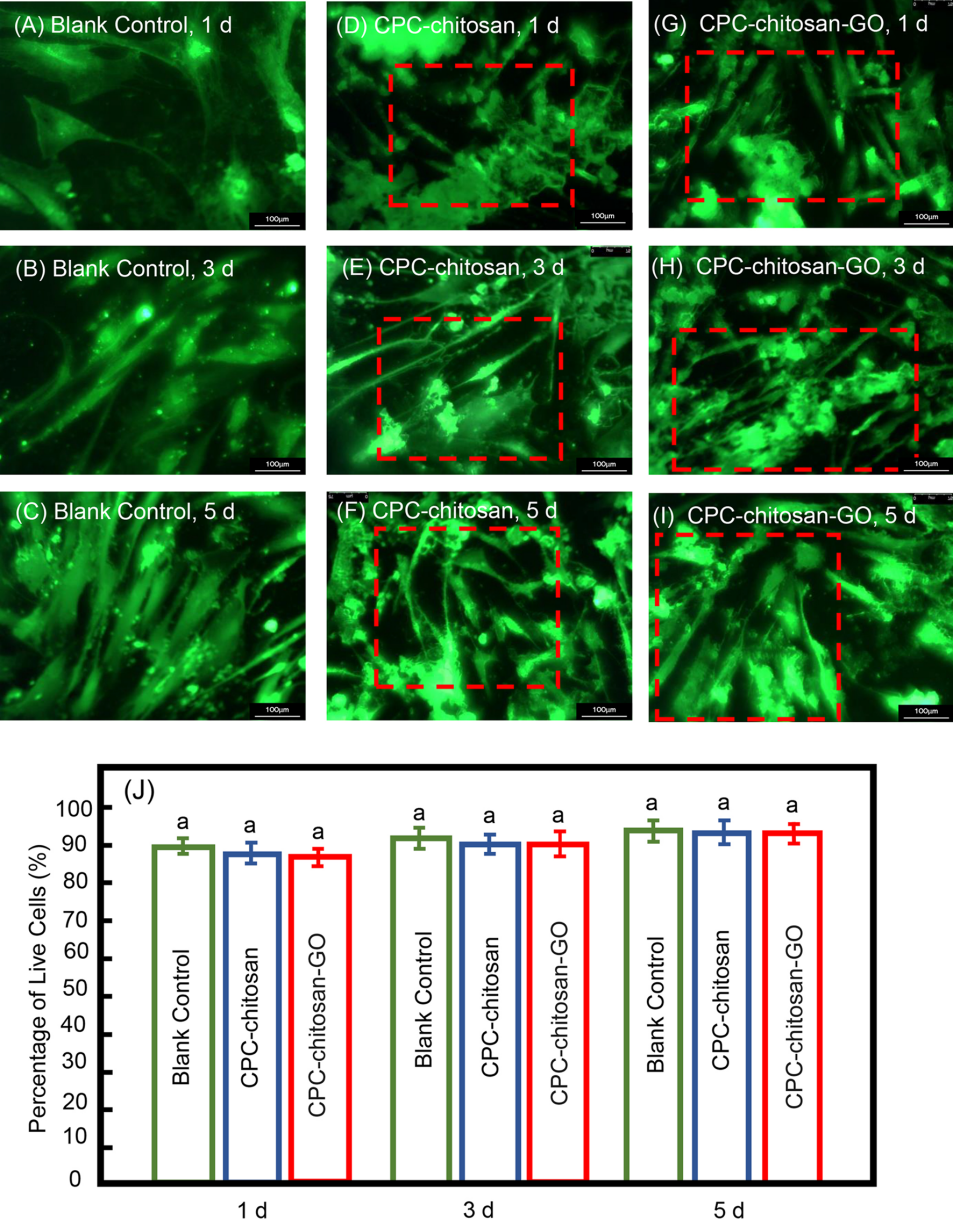
The viability of hDPSCs seeded on CPC-chitosan (**D**–**F**) or CPC-chitosan-GO disks (**G**–**I**) at 1 day, 3 days, and 5 days, respectively. Live cells (shown in green) were numerous. Dead cells (shown in red) were few. Compared with blank control (**A**–**C**) group, the cells were spread well on both CPC-chitosan and CPC-chitosan-GO group at 1 day, respectively. Gradually, the inter-laces among the cells and extracellular matrix based on both CPC-chitosan and CPC-chitosan-GO group were constructed at 3 days and 5 days. **J** Percentage of live hDPSCs on CPC-chitosan or CPC-chitosan-GO at 1 day, 3 days, and 5 days, respectively. There was no significant difference between the CPC-chitosan group and CPC-chitosan-GO group (*p* > 0.1)

The live/dead staining images of the hDPSCs from 1 to 5 days are shown in Fig. 2A–I. The healthy cells spread on both CPC-chitosan and CPC-chitosan-GO scaffold, respectively. Large amounts of live cells in green color while few red-stained dead cells were detected in both groups. In Fig. 2J, there was no significant difference between the two groups (*p* > 0.1), and the percentages of live cells on CPC-chitosan with or without GO powder were around 90%, indicating the existence of GO powder did not affect the hDPSC viability. The cells appeared to be well attached and extended on the surface of CPC-chitosan and CPC-chitosan-GO disks, demonstrating that the CPC-chitosan-GO is biocompatible and supports hDPSC attachment, similar with CPC-chitosan scaffold.

### Antibacterial effects inhibiting *E. faecalis*

**Fig. 3:**
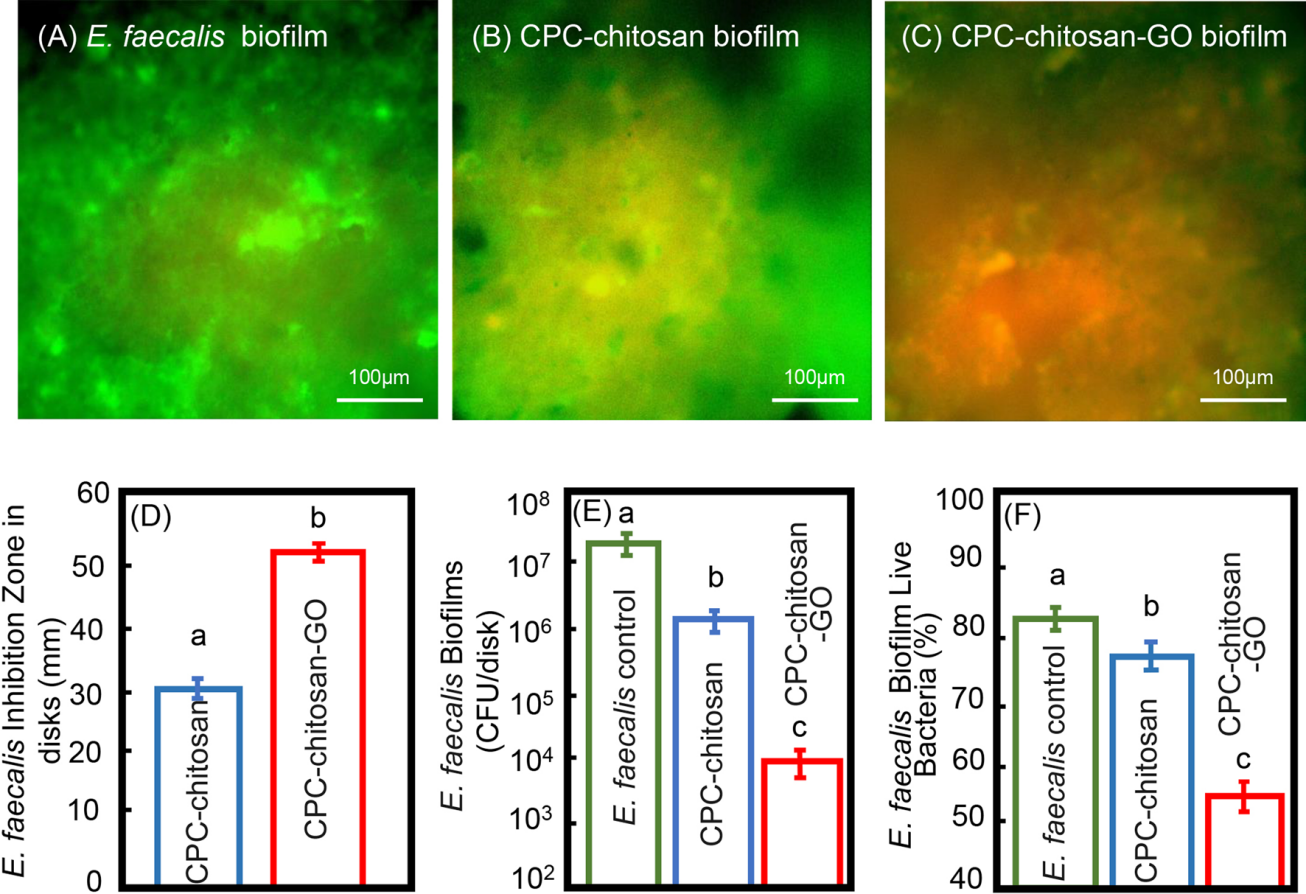
The antibacterial effects of CPC-chitosan scaffold and CPC-chitosan-GO disks were measured. Live and dead assay of bacterial biofilms on CPC-chitosan scaffold and CPC-chitosan-GO disks at 24 h. *E. faecalis* biofilms were served as control: The control group (**A**) and CPC-chitosan group (**B**) were covered by live bacteria. The CPC-chitosan-GO group (**C**) had more dead bacteria with red staining. Scale bar = 100 μm. **D** The inhibition zone size of CPC-chitosan decreased as compared with CPC-chitosan-GO (mean ± sd; n = 10). **E** CPC-chitosan-GO group demonstrated much lower biofilm CFU, compared to CPC-chitosan group (*p* < 0.05). **F** The percentage of live *E. faecalis* on CPC-chitosan scaffold and CPC-chitosan-GO disks. Dissimilar letters indicate significantly different values (*p* < 0.05)

The CPC-chitosan control group had more attached live bacteria than CPC-chitosan-GO group (Fig. 3A–C). The diameters of inhibition zone were measured in the CPC-chitosan and CPC-chitosan-GO group, respectively. The diameter of inhibition zones in CPC-chitosan-GO group was about 1.7 times that in CPC-chitosan group at 24 h (*p* < 0.05, Fig. 3D). The CFU counts for 24-h *E. faecalis* biofilms in each group were shown in Fig. 3E. CPC-chitosan-GO groups had CFU counts that were 2 logs lower than those of the CPC-chitosan group (*p* < 0.05). Quantitatively, the proportion of viable *E. faecalis* was 55.8 ± 4.8% in CPC-chitosan-GO group which was much lower than that in the control group (83.0 ± 3.8%) or CPC-chitosan group (76.2 ± 4.2%), respectively (*p* < 0.05, Fig. 3F).

**Fig. 4:**
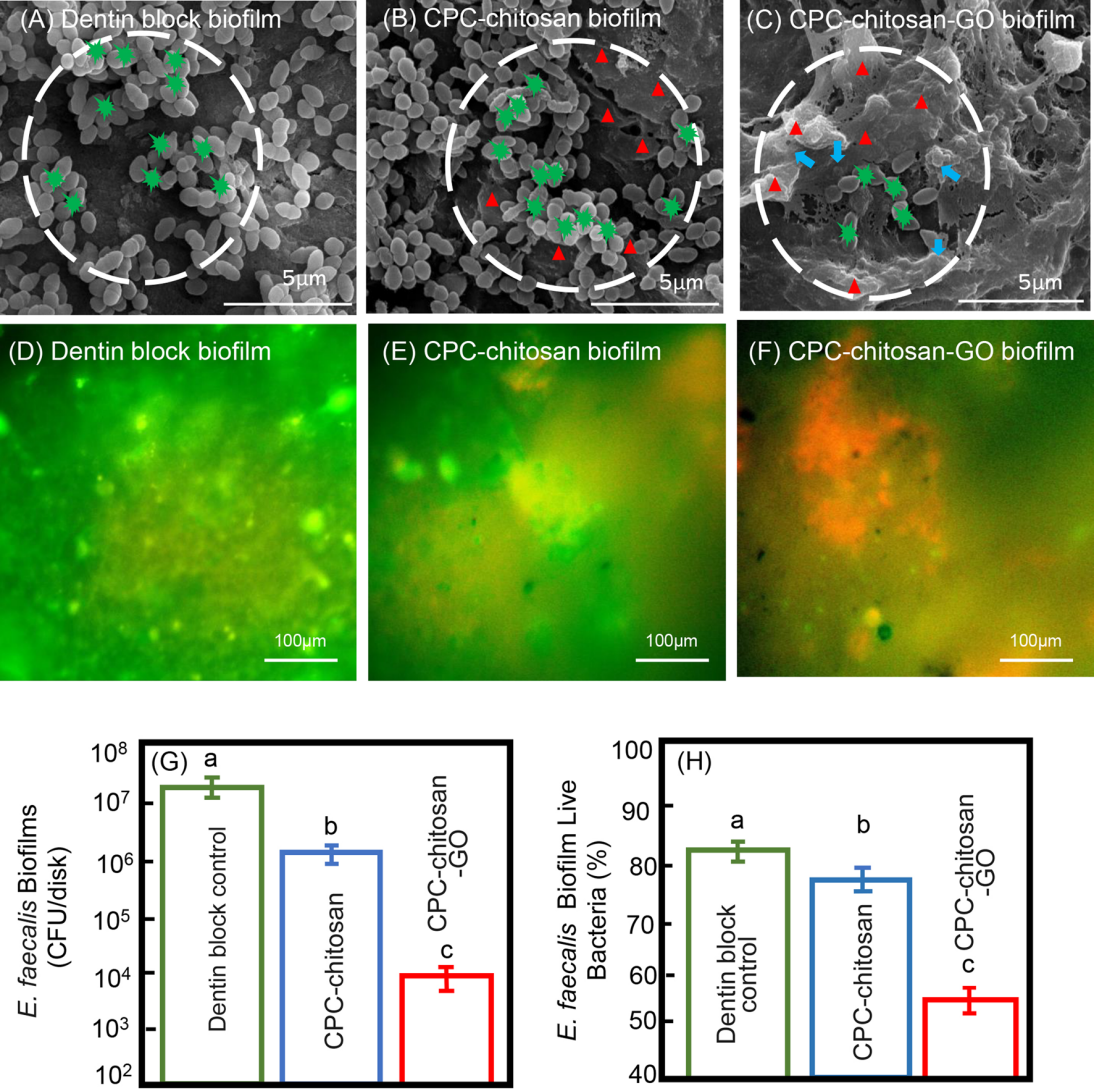
A clinical isolate of *E. faecalis* were used for comparison. Surface characteristics of CPC-chitosan-GO scaffold and antibacterial effects of CPC-chitosan-GO disks on *E. faecalis* clinical isolate: **A** The dentin control group. **B** There were *E. faecalis* colonies spreading among the interlaced CPC-chitosan, and **C** there were few *E. faecalis* cells spreading on CPC-chitosan-GO. The green stars show E. faecalis colonies, the red triangles show the CPC-chitosan composites and the blue arrows indicate GO powder. Live and dead assay of bacterial biofilms on CPC-chitosan scaffold and CPC-chitosan-GO disks at 24 h (**D**–**F**). Clinical isolate *E. faecalis* biofilms were served as control (D), CPC-chitosan group (**E**) were covered by live bacteria, and the CPC-chitosan-GO group (**F**) had more dead bacteria with red staining. **G** CPC-chitosan-GO group demonstrated much lower biofilm CFU, compared to CPC-chitosan group (*p* < 0.05). **H** The percentage of live *E. faecalis* on CPC-chitosan scaffold and CPC-chitosan-GO disks. Dissimilar letters indicate significantly different values (*p* < 0.05)

For further verification, the dentin block samples (Fig. 4A) and clinical isolates *E. faecalis* strains were involved. In CPC-chitosan group, there were obvious *E. faecalis* colonies (green star) growing among the CPC-chitosan composites (red triangle) (Fig. 4B). Compared with CPC-chitosan group, only little *E. faecalis* colonies were observed adhering to the surface of the interlaced CPC-chitosan paste mixed with GO powder (blue arrow) in CPC-chitosan-GO dentin samples (Fig. 4C). The biofilm vitality assays showed that the CPC-chitosan control group had more attached live bacteria than CPC-chitosan-GO group (Fig. 4D–F). Consistently, the CFU counting and quantitative viable proportion analysis demonstrated that CPC-chitosan-GO group had the lowest viable *E. faecalis* cells (*p* < 0.05, Fig. 4G, H).

## Discussion

In the present study, an injectable CPC-chitosan-GO antibacterial scaffold was developed. Depending on the existence of GO nano particles, it possesses a potent antibacterial ability against *E. faecalis* which is a major pathogen (36.6%) contributing to failed root canal therapy, and having potential for root canal sealer Also, it may be applied for periodontal repair and regeneration combined with bacterial infections. The endodontic treatment strategies involve infections control and the pathogenic tissue removal as well as prevention of reinfection and periapical lesions [19]. Although the root canal preparation can mostly reduce the number of bacteria, these procedures are not effective enough to eliminate residual bacteria because of the anatomical complexity of the root canal system [3]. *E. faecalis* could resist to the amenable situation in root canal system and cause reinfection [20]. Therefore, enhancing the antibacterial properties, biocompatibility, manipulating ability, and physical properties of root canal sealers may improve the successful rate of endodontic therapy [21].

Recently, calcium phosphate cements (CPC), a kind of good biocompatibility, osseointegration and osteo-conduction tissue engineering material, has been widely used in both orthopedics and dentistry fields including filling bone defects and vital pulp therapy [22]. CPC can be used as an injectable scaffold biomaterial for bones regeneration, to regenerate dental pulp or tissues in dentistry applications or engineering [23]. Previous studies examined the CPC scaffold in scanning electron microscope (SEM), showing high pore volume fraction, including intrinsic pores and nano-sized apatite minerals like those in natural bone [24]. The stem cells attached well to this bone mineral-mimicking CPC scaffold [25]. Our SEM results showed CPC-chitosan-GO disk had similar surface morphology with CPC-chitosan disk and GO nano particles spread inside the disk.

The CPCs could provide limited antimicrobic capacity for bone infections by impregnated with antibiotics [26]. However, there has been a continuing appearance of antibiotic-resistant strains detected in infected periapical tissues [27]. Graphene oxide as a kind of antibacterial nanoparticle, includes advantages of low toxicity, overcome resistance and high biocompatibility. Therefore, it plays a promising role on nanoplatform with the potential for novel antibacterial strategies particularly for multidrug-resistance bacteria [28]. In this study, our results indicated CPC-chitosan-GO had an excellent antimicrobial effect compared with CPC-chitosan scaffold. The main antibacterial mechanism of GO might probably attribute to the physical sharp edge as “nano-knife” puncturing and damaging the bacterial membranes which resulted in sequent reactional oxidative stress. Also, GO could restrict the growth of microorganisms by trapping the bacteria from their environment [28]. In CPC-chitosan-GO disks, the GO particle could release from them when immersed with *E. faecalis* suspension and inhibit the growth and viability of *E. faecalis* due to the above mechanisms.

Considering the clinical applications, our injectable systems could adjust paste to the defect surfaces and shape in situ for dental and craniofacial reconstructions with the minimal access. Also, the use of defensive antibacterial coating (DAC) has been reported to be easily applied in on the grafts, during anterior cruciate ligament reconstruction [29]. Previous studies reported that CPC, even containing pyrogens and chopped fibers, could be rendered injectable via a 10-gauge needle [30]. In this study, an injectable CPC-chitosan-GO paste could be applied as a filler/sealer for endodontic defect with favorable injectability, simple manipulation, and good antibacterial activity.

Currently, the stem cell-based therapies represent the most promising tool for successful regeneration of pathological dental tissues [31]. Pulpal mesenchymal stem cells (MSCs) are the first type of dentoalveolar tissues derived MSCs isolated from adult human dental pulpal tissues [32]. Unlike hBMSCs as a gold-standard of stem cell research, the hDPSCs can be collected from the extracted teeth without invasive methods and more likely to generate a pulp/dentin-like complex containing odontoblastic cells and vascularized fibrous tissue than hBMSCs [33].

The combination of stem cells and biomaterials can significantly improve regeneration effect [34]. Our previous studies showed both hDPSCs and hBMSCs could present an excellent viability, odontogenic differentiation, and mineralization in CPC-chitosan scaffold [15]. In this study, the vital stem cell ratios were similarly well performed in both groups. Therefore, it was speculated that GO with a concentration of lower than 50 μg/mL has no adverse effect on biocompatibility of CPC-chitosan scaffold. Next, the periapical periodontitis would be induced in the animal experiments, and the histological examinations and micro-CT analyses would be applied to evaluate the periapical lesions. To justify clinical application of the CPC-chitosan-GO material, the clinical symptoms and healing of periapical bone would be assessed in further investigations.

## Conclusions

CPC-chitosan-GO scaffold yielded excellent hDPSC viability and supported hDPSC attachment and growth. Furthermore, the novel CPC-chitosan-GO scaffold exhibited excellent antibacterial effects against *E. faecalis*. Therefore, CPC-chitosan-GO paste is promising for dental applications as root canal sealer to control infections and support stem cell viability for endodontic tissue regeneration.

## Data Availability

All data generated or analyzed during this study are included in this published article and its supplementary information files.

## Abbreviations

*E. faecalis*: *Enterococcus faecalis*
hDPSCs: Human dental pulp stem cells
SEM: Scanning electron microscopy
CLSM: Confocal Laser Scanning Microscope
GO: Graphene oxide
